# Supplementary material for: Correlation between Polymerase Chain Reaction Identification of Iron Acquisition Genes and an Iron-Deficient Incubation Test for Klebsiella pneumoniae Isolates from Bovine Mastitis
Source: Microorganisms. 2022 May 31;10(6):1138. doi: 10.3390/microorganisms10061138 (PMC9228167; doi:10.3390/microorganisms10061138)
Supplement: Supplementary file 1 [file microorganisms-10-01138-s001.zip › Table S1.pdf]

Table S1. Clinical data and milk conditions in bovine cases of mild mastitis caused by intramammary infections with *Klebsiella pneumoniae* tested in this study.

Isolate	Farm	Age (days) at exam	Days since calving at exam	Parity at exam	Mastitis milk conditions		Interval (days) between exam and death	Cause of the death
					Bacterial counts[1]	Score [22]		
#1	A	1330	17	2	8.28	1	2277	Culling (mastitis)
#2	A	1378	296	2	2.30	3	541	Culling (mastitis)
#3	A	1794	50	3	7.00	3	1056	Culling (low reproductive performance)
#4	A	3006	21	5	4.95	3	662	Culling (mastitis)
#5	A	990	3	1	7.85	2	1493	Death (unknown reason)
#6	A	2741	11	6	4.00	1	36	Culling (unknown reason)
#7	A	2585	2	5	5.54	2	1565	Culling (low reproductive performance)
#8	A	2479	233	4	4.70	3	605	Culling (low reproductive performance)
#9	B	2167	228	4	7.00	2	263	Culling (planned for low productivity)
#10	B	1297	73	2	7.30	2	1031	Culling (lameness)
#11	B	1479	4	3	5.34	4	664	Culling (mastitis)
#12	B	1245	214	2	4.85	1	411	Culling (lameness)
#13	C	2540	211	5	6.78	2	149	Culling (low reproductive performance)
#14	C	2281	192	4	4.48	3	93	Culling (planned for low productivity)
#15	C	2849	142	5	7.30	2	Unknown	Unknown

#16	C	1667	243	3	9.74		1033	Death (unknown reason)
#17	D	3371	213	7	8.18	2	151	Sold
#18	D	2202	340	3	3.48	2	445	Sold
#19	D	1214	72	2	2.60	3	314	Culling (mastitis)
#20	E	2396	197	5	9.14	1	535	Culling (mastitis)
#21	E	3007	218	6	7.30	2	403	Culling (mastitis)
#22	E	2896	100	6	4.14	4	Unknown	Unknown
#23	F	1628	297	2	7.39	1	610	Death (unknown reason)
#24	F	1922	14	4	6.70	3	147	Culling (mastitis)
#25	G	1580	Unknown	Unknown	3.00	1	1045	Culling (unknown reason)
#26	G	1215	Unknown	Unknown	8.18	2	80	Culling (unknown reason)
#27	H	2918	642	Unknown	5.30	4	89	Culling (unknown reason)
#28	I	789	50	1	4.70	2	541	Culling (mastitis)
#29	J	Unknown	Unknown	Unknown	6.37	3	Unknown	Unknown
#30	K	2287	514	3	2.30	3	21	Sold
#31	L	1810	93	3	4.60	4	Unknown	Unknown
#32	M	2950	364	Unknown	6.41	1	243	Culling (unknown reason)

#33	N	2033	2	4	8.28	3	1312	Culling (planned for low productivity)
#34	O	Unknown	Unknown	Unknown	6.34	2	Unknown	Unknown
#35	P	1714	Unknown	Unknown	4.18	4	559	Culling (unknown reason)
#36	Q	1351	136	2	7.00		Unknown	Unknown
#37	R	919	3	1	3.66	4	845	Sold

[1] Milk bacterial counts of *Klebsiella pneumoniae* are shown by $\log_{10}$ colony-forming units/ml.
