# Supplementary material for: Correlation between Polymerase Chain Reaction Identification of Iron Acquisition Genes and an Iron-Deficient Incubation Test for Klebsiella pneumoniae Isolates from Bovine Mastitis
Source: Microorganisms. 2022 May 31;10(6):1138. doi: 10.3390/microorganisms10061138 (PMC9228167; doi:10.3390/microorganisms10061138)
Supplement: Supplementary file 1 [file microorganisms-10-01138-s001.zip › Table S2.pdf]

Table S2. Primers used in this study.

Primer name	Sequence (5'- 3')	Size of product	References
K1-F	GGTGCTCTTTACATCATTGC	1283	[9]
K1-R	GCAATGGCCATTTGCGTTAG		
K2-F	GACCCGATATTCATACTTGACAGAG	641	[9]
K2-R	CCTGAAGTAAAATCGTAAATAGATGGC		
*mag*A-F	GGTGCTCTTTACATCATTGC	1280	[9,13]
*mag*A-R	GCAATGGCCATTTGCGTTAG		
*rmp*A-F	ACTGGGCTACCTCTGCTTCA	536	[9]
*rmp*A-R	CTTGCATGAGCCATCTTTCA		
*iuc*A-F	GCATAGGCGGATA CGAACAT	556	[9]
*iuc*A-R	CACAGGGCAATTG CTTACCT		
*ent*B-F	TGAAGACGATACCGTGCTGGTGGA	371	[14]
*ent*B-R	GTCGGCGACAAAGAACGGTTTGAT		
*fep*A-F	CGACGTCTCGGAGATCATT	554	[5]
*fep*A-R	GATATCAATCTGGCGGTTGTT		
*ybt*S-F	CAAAAATGGGCGGTGGATTC	242	[14]
*ybt*S-R	CCTGACGGAACATAAACGAGCG		

psn-F	CAAACTCCCCAGAGTCTTGC	1062	[5]
psn-R	TAGAAGTCCTGCGCTGAAGA		
kfu-F	GAAGTGACGCTGTTTCTGGC	797	[9,13]
kfu-R	TTTCGTGTGGCCAGTGACTC		
