# Supplementary material for: Correlation between Polymerase Chain Reaction Identification of Iron Acquisition Genes and an Iron-Deficient Incubation Test for Klebsiella pneumoniae Isolates from Bovine Mastitis
Source: Microorganisms. 2022 May 31;10(6):1138. doi: 10.3390/microorganisms10061138 (PMC9228167; doi:10.3390/microorganisms10061138)
Supplement: Supplementary file 1 [file microorganisms-10-01138-s001.zip › Table S3.pdf]

Table S3. Polymerase chain reaction (PCR) detection[1] of six iron-acquisition genes in *Klebsiella pneumoniae* isolates tested in this study.

Isolate	Farm	*iuc*A	*ent*B	*fep*A	*ybt*S	*psn*	*kfu*	Number of the genes detected by PCR
#1	A	-	-	-	-	-	-	0
#2	A	-	-	-	-	-	-	0
#3	A	-	-	-	-	-	-	0
#4	A	-	-	-	-	-	-	0
#5	A	-	-	+	-	-	-	1
#6	A	-	+	+	-	-	+	3
#7	A	-	+	+	+	+	-	4
#8	A	-	+	+	+	+	+	5
#9	B	-	+	-	-	-	-	1
#10	B	-	-	+	-	-	+	2
#11	B	-	+	+	-	-	-	2
#12	B	-	+	+	-	-	-	2
#13	C	-	-	+	-	-	-	1
#14	C	-	+	+	-	-	-	2
#15	C	-	+	+	-	-	-	2
#16	C	-	+	+	-	-	+	3

#17	D	-	+	-	-	+	-	2
#18	D	-	+	+	-	-	-	2
#19	D	-	+	+	-	-	-	2
#20	E	-	-	+	-	-	-	1
#21	E	-	+	+	-	-	-	2
#22	E	-	+	+	-	-	+	3
#23	F	-	-	-	-	-	-	0
#24	F	-	+	+	-	-	+	3
#25	G	-	-	+	-	-	+	2
#26	G	-	+	+	-	-	-	2
#27	H	-	-	-	-	-	-	0
#28	I	-	-	-	-	+	-	1
#29	J	-	+	-	-	-	-	1
#30	K	-	+	-	-	-	+	2
#31	L	-	+	+	-	-	-	2
#32	M	-	+	+	-	-	-	2
#33	N	-	+	-	-	+	+	3

#34	O	-	+	+	-	-	+	3
#35	P	-	+	+	-	+	+	4
#36	Q	-	+	+	+	+	+	5
#37	R	-	+	+	+	+	+	5
Proportion[2] (%)		0	67.6	67.6	10.8	21.6	35.1	2.6 (1.7)[3]

[1] + and -, Each iron-acquisition gene is detected or not detected by the PCR test, respectively.

[2] Proportion of the isolates with PCR-detection of each iron-acquisition gene, out of 37 strains tested in this study.

[3] Mean (standard deviation) of PCR-detection numbers of iron-acquisition genes in 37 isolates.
