# Supplementary material for: Correlation between Polymerase Chain Reaction Identification of Iron Acquisition Genes and an Iron-Deficient Incubation Test for Klebsiella pneumoniae Isolates from Bovine Mastitis
Source: Microorganisms. 2022 May 31;10(6):1138. doi: 10.3390/microorganisms10061138 (PMC9228167; doi:10.3390/microorganisms10061138)
Supplement: Supplementary file 1 [file microorganisms-10-01138-s001.zip › Table S4.pdf]

Table S4. Association between bacterial counts [means (standard deviations); log_{10} colony-forming units/ml] and polymerase-chain-reaction (PCR)-positive iron-acquisition genes in *Klebsiella pneumoniae* isolates incubated in iron-half-sufficient medium.

Gene	PCR[1]	Day 0	Day 1	Day 2	Day 3
*ent*B	+	5.46 (0.20)[a]	7.65 (0.85)[b]	8.11 (0.74)[b]	8.11 (0.83)[b]
*ent*B	-	5.55 (0.32)[a]	7.71 (1.10)[b]	7.89 (0.81)[b]	7.80 (0.58)[b]
fepA	+	5.47 (0.25)[a]	7.70 (0.82)[b]	8.04 (0.82)[b]	8.12 (0.73)[b]
fepA	-	5.53 (0.23)[a]	7.58 (1.17)[b]	8.02 (0.65)[b]	7.78 (0.82)[b]
*ybt*S	+	5.52 (0.12)[a]	7.70 (0.98)[b]	8.38 (0.58)[b]	8.59 (0.29)[b]
*ybt*S	-	5.49 (0.26)[a]	7.66 (0.93)[b]	7.99 (0.78)[b]	7.94 (0.78)[b]
psn	+	5.54 (0.18)[a]	7.90 (0.87)[b]	8.18 (0.77)[b]	8.15 (0.74)[b]
psn	-	5.48 (0.26)[a]	7.60 (0.94)[b]	7.99 (0.77)[b]	7.97 (0.78)[b]
kfu	+	5.47 (0.27)[a]	7.93 (0.92)[b]	8.04 (0.85)[b]	8.08 (0.88)[b]
kfu	-	5.50 (0.24)[a]	7.52 (0.92)[b]	8.03 (0.73)[b]	7.97 (0.71)[b]
Total		5.49 (0.25)[a]	7.67 (0.92)[b]	8.03 (0.76)[b]	8.01 (0.76)[b]

[1] + and -, Each iron-acquisition gene is detected or not detected by the PCR test, respectively.

[a,b] Within row (among 0–3 incubation days) for each incubation group, numbers with different superscripts are significantly different ($p < 0.05$).
