# Supplementary figures and images for: Correlation between Polymerase Chain Reaction Identification of Iron Acquisition Genes and an Iron-Deficient Incubation Test for Klebsiella pneumoniae Isolates from Bovine Mastitis
Source: Microorganisms. 2022 May 31;10(6):1138. doi: 10.3390/microorganisms10061138 (PMC9228167; doi:10.3390/microorganisms10061138)

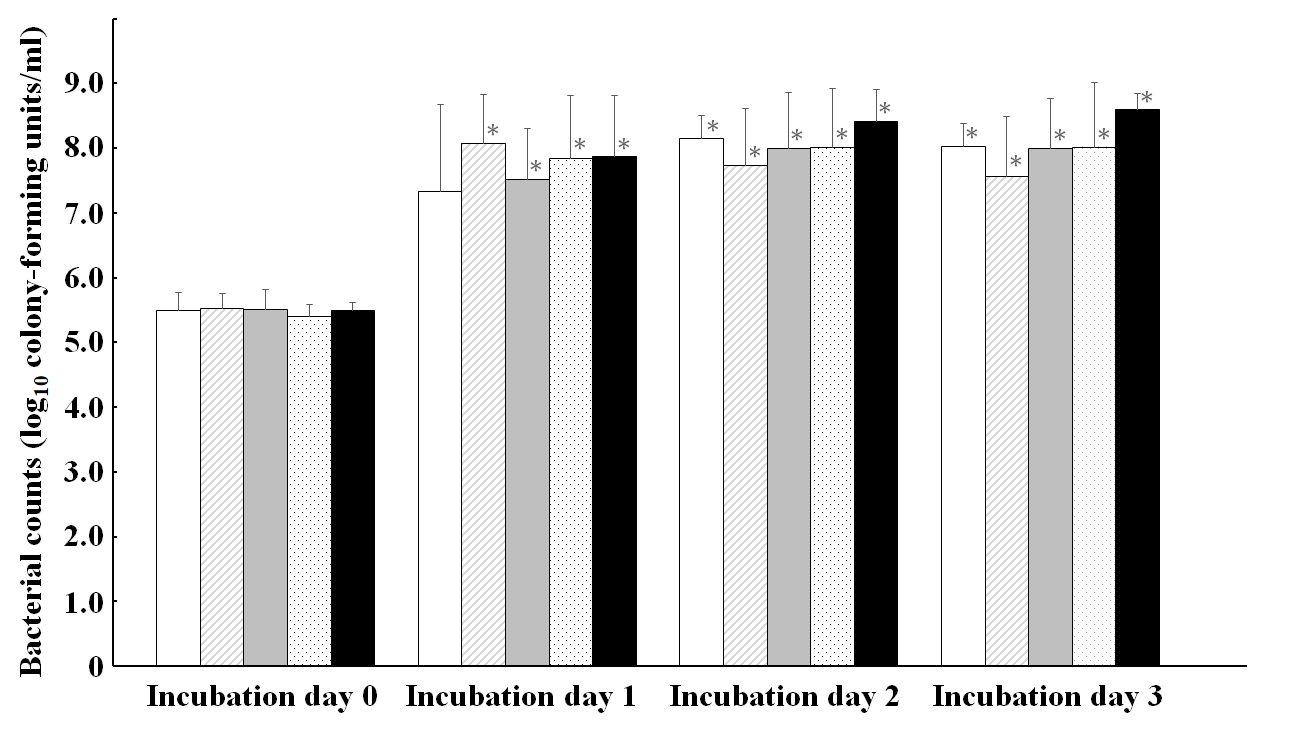

Supplement: Supplementary file 1 [file microorganisms-10-01138-s001.zip › Figure S1.tif]
